# Presence of cerebral microbleeds is associated with cognitive decline in acromegaly

**DOI:** 10.3389/fonc.2022.948971

**Published:** 2022-11-24

**Authors:** Zhengxing Xie, Yan Zhuang, Zongqiang Zhang, Jieping Liu

**Affiliations:** ^1^ Department of Neurosurgery, The Affiliated Hospital of Jiangsu University, Zhenjiang, China; ^2^ Neuro-Endoscope and Mini-Invasive Treatment Center, The Affiliated Hospital of Jiangsu University, Zhenjiang, China; ^3^ Department of Neurosurgery, Wuxi No. 2 People’s Hospital, Wuxi, China; ^4^ Department of Neurosurgery, Bingtuan Sishi Hospital, Yining, China

**Keywords:** acromegaly, growth hormone-secreting pituitary adenoma, microbleeds, cognitive decline, obstructive sleep apnea

## Abstract

**Background:**

Cognitive decline in acromegaly has gained increasing attention. Cerebral microbleeds (CMBs) as radiographic markers for microvascular injury have been linked to various types of cognitive decline. However, the association between CMB formation and acromegaly has not yet been quantified. This study is designed to investigate the prevalence and the radiographic patterns of CMBs and the association between cognitive function and acromegaly-related CMBs in growth hormone (GH)-secreting pituitary adenoma, which is characterized by acromegaly.

**Methods:**

In a cohort of 55 patients with GH-secreting pituitary adenoma (acromegaly) and 70 healthy control (HC) patients, we determined the presence of CMBs using a 3.0-T MRI scanner. The numbers, locations, and grades of CMBs were determined *via* susceptibility-weighted imaging (SWI) and the Microbleed Anatomical Rating Scale. Obstructive sleep apnea (OSA) was assessed using the criteria of the American Academy of Sleep Medicine (AASM) Scoring Manual Version 2.2. The Montreal Cognitive Assessment (MoCA) was used to assess cognitive performance within 3 days of admission. The association between CMBs and cognitive function as well as clinical characteristics was explored.

**Results:**

The incidence of CMBs was 29.1%, whereas that of OSA was 65.5% in acromegaly. There was a statistically significant difference in the prevalence of CMBs between subjects with and without acromegaly (29.1% and 5.3%, respectively) (*p* < 0.01). The age of acromegaly patients with CMBs was much younger compared with HCs with CMBs. Compared with HCs, a significant cognitive decline and the occurrence of OSA were demonstrated in patients with acromegaly (*p* < 0.01). Binary logistic regression analysis adjusted for age, education, and body mass index (BMI) revealed that CMB was an independent risk factor for cognitive impairment in patients with acromegaly (OR = 3.19, 95% CI 1.51–6.76, *p* = 0.002). Furthermore, in the logistic regression models adjusted for age, BMI, diabetes, and hypertension, OSA was independently associated with the occurrence of CMBs in patients with acromegaly (OR = 13.34, 95% CI 3.09–57.51, *p* = 0.001).

**Conclusions:**

A significant increase of CMBs was demonstrated in patients with acromegaly, which may be a result of OSA in acromegaly. The present study indicated that increasing CMBs are responsible for cognitive decline in patients with acromegaly.

## Introduction

Acromegaly, mostly due to a somatotroph pituitary adenoma, is an endocrine and metabolic disease characterized by chronic exposure to excess growth hormone (GH) and insulin-like growth factor 1 (IGF-1) levels (1, 2). The manifestation of acromegaly includes facial and acral changes as well as cardiorespiratory complications. Moreover, multiple adverse effects of acromegaly on systemic complications such as metabolic and oncologic complications have also been documented (3). Recently, cognitive deterioration has gained enormous attention in patients with acromegaly (4, 5). As we all know, physiologic GH and IGF-1 levels have demonstrated multiple important roles in the nervous system including brain growth, development, and myelination as well as neurogenesis processes and plasticity (6). However, the effect of chronic and persistent exposure to excess GH and IGF-1 on the brain is far from being understood. To date, the underlying biological mechanisms leading to acromegaly-associated neurocognitive deficits remain unclear. Whether such cognitive decline in acromegaly is a result of excessive secretion of GH and IGF-1 remains to be elucidated.

Cerebral microbleeds (CMBs) are neuroimaging markers of cerebral small vessel disease characterized by small and round lesions with clear margins ranging from 2 to 10 mm in size on susceptibility-weighted imaging (SWI). Growing evidence demonstrated that CMBs increased the risk of cerebrovascular and neurodegenerative pathology and led to stroke, cognitive dysfunction, and even dementia (7, 8). However, to date, there are no studies specifically focusing on the effect of CMB lesions on patients with acromegaly.

The purpose of the current study was to explore the prevalence of CMB in patients with acromegaly and its possible role in cognitive impairment in acromegaly. Furthermore, we explored the possible underlying mechanisms of the occurrence of CMBs in acromegaly.

## Methods

### Patients

After receiving ethics committee approval, we retrospectively analyzed our series of consecutive patients with growth hormone-secreting pituitary adenoma admitted at Wuxi No. 2 People’s Hospital and The Affiliated Hospital of Jiangsu University from February 2017 to June 2022. Age-, sex-, and education-matched HCs were recruited *via* social media and volunteers. Patients with a history of stroke, cerebral trauma, other diseases with cognitive disorder, other brain lesions, taking cognitive-affecting drugs within 1 week, drug or alcohol abuse, and general contraindication to MRI examinations scan were excluded.

Acromegaly was diagnosed according to the criterion established by the Endocrine Society and the American Association of Clinical Endocrinologists guidelines. Biochemical evidence of acromegaly was established with increased serum GH and IGF-I levels and/or nadir serum GH >0.4 μg/L post-oral glucose tolerance test (OGTT) (9). A pituitary magnetic resonance imaging (MRI) was performed to assess the evidence of adenoma, followed by pathological examination after surgery. Obstructive sleep apnea (OSA) was diagnosed according to the criteria of the American Academy of Sleep Medicine (AASM) Scoring Manual Version 2.2 (10). The apnea-hypopnea index (AHI) ≥5/h was accepted as OSA. The severity of OSA was classified as follows: mild (5 ≤ AHI < 15), moderate (15 ≤ AHI < 30), and severe (AHI ≥ 30).

### Clinical characteristics

All the patients’ records, radiological images, endocrine evaluation, and operative notes were reviewed, including gender, body mass index (BMI), age, years of education, serum GH (ng/ml), IGF-1 (ng/ml), and nadir serum GH post-OGTT, as well as evidence of OSA. Disease duration was determined by the earliest physical/somatic signs based on the patient’s history. Cognitive performance was evaluated using the Montreal Cognitive Assessment (MoCA) (11).

### MRI acquisition and processing

All subjects were scanned on a 3.0-T Siemens Prisma MRI scanner (Siemens Healthcare, Erlangen, Germany). It typically included axial T1-weighted [repetition time (TR), 2,100 ms; echo time (TE), 2.5 ms; flip angle (FA), 7°] images with a field of view (FOV) and matrix size of 256 × 256 mm^2^ and acquisition voxel size of 1.0 × 1.0 × 1.0 mm^3^, T2-weighted images (TR, 5,500 ms; TE, 120 ms) with an FOV and matrix size of 240 × 240 mm^2^ and acquisition voxel size of 1.0 × 1.0 × 5.0 mm^3^, and gadolinium-DTPA (Gd-DTPA) injection-enhanced (0.1 mmol/kg) axial T1-weighted images (TR, 450 ms; TE, 15 ms; section thickness, 5 mm) with an FOV and matrix size of 240 × 240 mm^2^. The parameters of the SWI sequence were as follows: TR = 27 ms, TE = 20 ms, FA = 15°, and slice thickness = 1.5 mm.

### Image analysis

Two experienced radiologists, blinded to clinical data, reviewed the MR images. CMBs are defined as focal, small, round to ovoid punctuate areas of hypointensity on SWI images, which range from 2 to 10 mm in size. The presence and number of definite CMBs were determined on SWI by the Microbleed Anatomical Rating Scale, which classifies CMBs according to topographic distribution in the brain and has demonstrated excellent intrarater and interrater reliability to map brain microbleeds in all brain locations when applied to different MRI sequences and levels of observer experience (12). Combined with number and location, we classified CMBs into three grades: mild (one CMB or two CMBs involved just one location), moderate (two CMBs involved two locations or multiple CMBs involved just one location), and severe (multiple CMBs involved ≥2 locations).

### Statistical analysis

Categorical variables were presented as *n* (%), while normally distributed variables were presented as mean (standard deviation). Group differences in nominal variables were tested using the chi-squared test. An unpaired *t*-test was applied to compare the continuous variables between groups. A two-tailed *P <*0.05 was considered statistically significant. Next, age, years of education, and variables with significant differences between groups with and without cognitive impairment in patients with acromegaly were adjusted in the following regression analysis. Binary logistic regression analysis was performed to determine whether the presence of CMBs was an independent risk factor of cognitive performance and whether the increase of OSA was an independent risk factor of CMBs. Statistical analysis for all data was performed using SPSS20 Statistics software.

## Results

### Clinical characteristics

Following the inclusion and exclusion criteria, 55 patients with acromegaly and 70 HC patients were enrolled in the study. No alterations in consciousness were found in all subjects. The mean age was 46.78 ± 10.01 years in patients with acromegaly including 25 men and 30 women and 48.27 ± 10.46 years in HCs including 30 men and 40 women. The mean BMI was 22.44 ± 2.34 in patients with acromegaly and 21.82 ± 2.16 in HCs. The demographic information and clinical characteristics of the participants are shown in **Table 1**. There were no significant differences between the two groups in terms of age, education level, and BMI. Twenty-five patients demonstrated MoCA scores less than 26 in acromegaly, while there were only 2 in HCs. Comparisons of the cognitive data between patients with acromegaly (23.20% ± 4.12%) and HCs (27.73% ± 1.19%) showed significant differences in terms of the MoCA (*p* < 0.01). Thirty-six patients demonstrated OSA (mild 15, moderate 9, severe 12) in the acromegaly group and 4 patients (mild 3, moderate 1) in the HC group. Significant differences were observed between the two groups in terms of OSA (*p* < 0.01). Diabetes was demonstrated in 35 patients with acromegaly and 28 patients showed hypertension.

**Table 1:** Demographics and clinical characteristics of study patients and healthy control subjects.

Characteristics	Acromegaly patient	HC	*p*-values
Case	55	70	
Diabetes	35	0	
Hypertension	28	0	
Age (mean ± SD, years)	46.78 ± 10.01	48.27 ± 10.46	0.42
Gender (male/female)	25/30	30/40	0.77
Education (years)	12.62 ± 3.88	13.05 ± 3.45	0.51
BMI	22.44 ± 2.34	21.82 ± 2.16	0.13
Estimated disease duration (months)	54.09 ± 31.76		
OSA	36	4	<0.01
Mild	15	3	
Moderate	9	1	
Severe	12	0	
MoCA (scores)	23.20 ± 4.12	27.73 ± 1.19	<0.01
<26	25	2	

### CMB characteristics in the study groups

**Figure 1:**
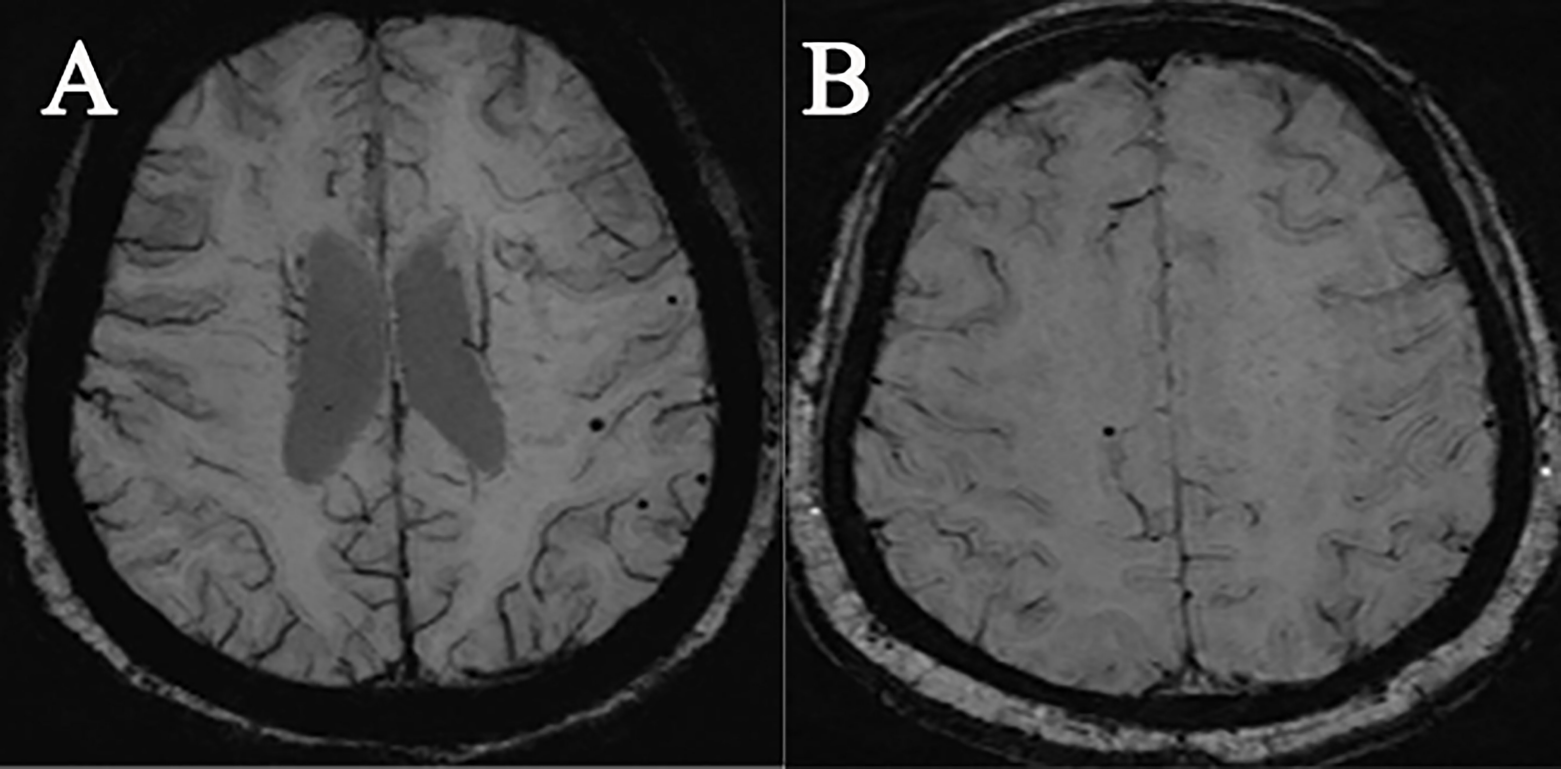
Demonstration of SWI images of two types of subjects with microbleeding: **(A)** a female acromegaly patient, 32 years old; **(B)** a female HC, 70 years old.

CMB characteristics in the two study groups are shown in **Table 2**. CMBs were demonstrated in 16 patients in the acromegaly group and in 4 patients in the HC group (**Figure 1**). Comparisons of the CMB data between patients with acromegaly (29.09%) and HCs (5.71%) showed significant differences (*p* = 0.001). Most CMBs were found in subjects less than 60 years old (93.75%) in the acromegaly group, while no CMBs were found in patients less than 60 years old in the HC group. The numbers of CMBs in the acromegaly group were much larger than those in the HC group. The regional brain distribution of CMBs was the highest in the lobar regions (87.5%) in the acromegaly group, with 4 patients having mixed-type CMBs in the deep brain region and about 10 having strictly lobar CMBs, while in the HC group, the distribution of CMBs was the highest in the deep brain regions (50%) and there were no mixed-type CMBs found. The distribution of CMBs is shown in **Table 2**. Based on the total population of study subjects, further analysis showed differences between the two groups concerning study subjects with moderate and severe CMBs (5.45% vs. 0%; 16.36% vs. 0%).

**Table 2:** CMB characteristics in the study groups.

Parameters	Acromegaly patient	HC	*p*-value
Total population	55	70	
Total population with CMBs	16	4	0.001
Frequency of multiple CMBs			
0 CMBs	39	66	
1 CMBs	3	4	
2 CMBs	4	0	
≥3 CMBs	9	0	
Population with CMBs (<60 years)	15	0	
Regional brain distribution of CMBs			
Lobar region	14	1	
Deep brain regions	6	2	
Infratentorial region	2	1	
Grade of CMBs			
Mild	4	4	
Moderate	3	0	
Severe	9	0	

### Role of CMBs in cognitive decline and associations between OSA and CMBs in acromegaly

In our study, we found that patients with acromegaly demonstrated more significant cognitive decline than HCs. Thus, we further analyzed whether there were potential associations between cognitive dysfunction and CMBs in acromegaly. Binary logistic regression analysis adjusted for age, education, and BMI revealed that CMBs were an independent risk factor for cognitive impairment in acromegaly (OR = 3.19, 95% CI 1.51–6.76, *p* = 0.002). The results of the logistic regression analysis for the other variables were as follows: education (OR = 0.69, 95% CI 0.19–2.51, *p* = 0.58), BMI (OR = 1.21, 95% CI 0.49–3.02, *p* = 0.68), and age (OR = 1.12, 95% CI 0.24–5.24, *p* = 0.88). In addition, we further investigated the potential mechanism of the occurrence of CMBs in acromegaly. In patients with acromegaly, the logistic regression models adjusted for age, BMI, diabetes, and hypertension demonstrated that OSA was independently associated with the increased prevalence of CMBs (OR = 13.34, 95% CI 3.09–57.51, *p* = 0.001). The results of the logistic regression analysis for the other variables were as follows: BMI (OR = 1.10, 95% CI 0.07–16.59, *p* = 0.94), age (OR = 1.21, 95% CI 0.07–20.69, *p* = 0.89), diabetes (OR = 18.23, 95% CI 0.48–680.44, *p* = 0.12), and hypertension (OR = 22.61, 95% CI 0.92–555.73, *p* = 0.06).

## Discussion

It has been well established that acromegaly characterized by excess GH and IGF-1 levels causes a systemic involvement with multiple comorbidities and increasing mortality in patients. However, little is documented about the effect of persistent excess GH and IGF-1 levels on brain alteration. Here, our analysis of 55 patients with acromegaly is the first to demonstrate that CMBs develop frequently in acromegaly and that the presence of CMBs is associated with MoCA scores in this population. This finding suggests that CMBs are important factors for cognitive impairment in acromegaly. Moreover, significantly positive correlations exist between increasing CMBs and OSA. We believe that OSA in acromegaly results in the significant occurrence of CMBs, consequently contributing to the cognitive decline in acromegaly.

CMBs are defined as hypointense foci visible on T2*-weighted and susceptible-weighted MRI (SWI) sequences which are characterized histologically by the presence of hemosiderin around small vessels (13). As a manifestation of cerebral small vessel pathologies, CMBs have been demonstrated in increasing diseases, such as stroke (14), traumatic brain injury (15), and chronic kidney disease (16). However, the prevalence of CMBs in acromegaly has not been documented. Here, we demonstrate that CMBs exist in patients with acromegaly with a higher prevalence of about 29.09% than in HCs, which suggests an injury of the cerebral small vessel after persistent exposure to excess GH and IGF-1 levels. Moreover, compared with HCs, the age of patients with CMBs in acromegaly is much younger. All these suggest the possible underlying role of CMBs in acromegaly.

CMBs are increasingly recognized as helpful markers of vascular pathology and hemorrhagic stroke (17). In addition to their role as markers of an underlying disease, growing evidence has demonstrated that CMBs could have direct effects on neurologic function, cognition, and disability (18–21). Evidence shows the association of CMBs with impairments in executive function and processing speed (22, 23). Indeed, neuropathological analyses of CMBs generally find these lesions to be associated with some degree of surrounding tissue damage offering a potential mechanism for brain dysfunction. Moreover, emerging evidence has demonstrated the role of genetic factors and gene–environmental interactions which might shed light on the underlying etiologies of CMBs (24).

Recently, considerable attention has been directed to the impairment of brain function in acromegaly (25). Some studies demonstrated that a considerable proportion of patients with acromegaly perform below average in several cognitive tests, particularly in the domains of attention and memory (5). Consistent with previous results, our study demonstrated that a significant cognitive decline exists in patients with acromegaly when compared with HCs. However, the neurobiological basis for such cognitive impairment in acromegaly is far from being understood. Previous evidence showed no associations of patients’ cognitive performance in acromegaly with their status of biochemical control or the presence of comorbidities such as diabetes mellitus or hypertension (4), which further supported that cognitive impairment represents a genuine pathological entity in acromegaly. Thus, despite the appearance of several studies about the underlying mechanism of cognitive impairment in acromegaly, these conclusions warrant further evidence to interpret cognitive decline. Considering the higher prevalence of CMBs in acromegaly and the contribution of CMBs to cognitive decline, we found a positive relationship between CMBs and cognitive decline in acromegaly. This is in concordance with previous reports that CMBs are associated with cognitive decline. Given the prevalence of CMBs in populations with dementia, our results support the concern that patients with acromegaly may experience accelerated aging. In our study, we find that there are more patients with severe grade CMBs in acromegaly, which is more susceptible to result in a decline of MoCA scores. We did not find the impact of one CMB on cognitive decline. The effect of CMBs on cognitive decline may be a recombination effect of both the number and location of CMBs. However, because of the limitation of our relatively small series, we cannot determine the cutoff value regarding the number of CMBs or the grade of CMBs, which requires more cases to elucidate such recombination effect and will be explored in our next study.

The critical role of GH/IGF-1 in brain growth and development has been well established; however, little is known about the effect of excessive GH/IGF-1 on the brain. Whether the underlying mechanism of the high prevalence of CMBs in acromegaly is a direct result of excess GH/IGF-1 dynamics or rather an indirect effect remains unclear. Acromegaly is associated with various systemic complications including hypertension and diabetes. The association between hypertension or diabetes and CMBs has been studied previously. However, no significant difference was demonstrated in exploring the possible association between hypertension or diabetes and CMBs in patients with acromegaly in our series. We suggest that two factors may contribute to such a result. First, either diabetes or hypertension has demonstrated a high prevalence in acromegaly patients, which may lead to statistical bias. Then, as we know, hypertension and diabetes are associated with CMBs in the deep brain region. In our series, most CMBs are found in the lobe region in acromegaly (14/16), which may also contribute to the result.

As we all know, the association between OSA and cerebral small vessel disease has been well established (26, 27). Moderate to severe OSA has been established as one of the independent predictors of CMBs (28, 29). Growing evidence has demonstrated a high prevalence of OSA in acromegaly (30). Consistent with previous studies, OSA was detected in 65.5% of active acromegaly patients in our study. Our result also demonstrated a positive relationship between CMBs and OSA, suggesting that OSA may provide an intriguing link between excess GH/IGF-1 and CMBs. We believe that OSA plays an important role in cognitive decline *via* the formation of CMBs in acromegaly. Our results are consistent with a previous study that poor sleep quality is associated with poorer cognitive function in acromegaly (31).

In conclusion, our findings demonstrated that long-term, persistent, and excess serum GH/IGF-1 levels result in OSA which helps explain the overlap between acromegaly and the higher prevalence of CMBs. As a result, such increasing CMBs may be responsible for the cognitive decline in acromegaly.

## Data availability statement

The original contributions presented in the study are included in the article/supplementary material. Further inquiries can be directed to the corresponding author.

## Ethics statement

The studies involving human participants were reviewed and approved by the Wuxi No. 2 Hospital Ethics Committee. The patients/participants provided their written informed consent to participate in this study.

## Author contributions

ZX designed and conceptualized the study. ZX, YZ, JL, and ZZ played a major role in the acquisition of data. ZX and YZ analyzed the data. ZX drafted the manuscript for intellectual content. All authors contributed to the article and approved the submitted version.

## Funding

This study was funded by the Talent Start-Up Fund of the Affiliated Hospital of Jiangsu University (No.jdfyRC2022001).

## Acknowledgments

We are very grateful to all the participants who volunteered to take part in the study.

## Conflict of interest

The authors declare that the research was conducted in the absence of any commercial or financial relationships that could be construed as a potential conflict of interest.

## Publisher’s note

All claims expressed in this article are solely those of the authors and do not necessarily represent those of their affiliated organizations, or those of the publisher, the editors and the reviewers. Any product that may be evaluated in this article, or claim that may be made by its manufacturer, is not guaranteed or endorsed by the publisher.
